# PREDICTORS OF PARTICIPATION IN A TELEPHONE-BASED SOCIAL CONNECTEDNESS INTERVENTION FOR OLDER ADULTS

**DOI:** 10.1093/geroni/igac059.929

**Published:** 2022-12-20

**Authors:** Omolola Adepoju, Ben King, Jiangtao Luo, LeChauncy Woodard, Jessica Dobbins, Jason Pierett, William Glasheen

**Affiliations:** University of Houston College of Medicine, Houston, Texas, United States; University of Houston College of Medicine, Houston, Texas, United States; University of Houston, Houston, Texas, United States; University of Houston College of Medicine, Houston, Texas, United States; Humana, Louisville, Kentucky, United States; Humana, Louisville, Kentucky, United States; Humana, Louisville, Kentucky, United States

## Abstract

**Background:**

Social isolation is a well-documented contributor to poor mental and physical health, and interventions promoting social connectedness have been shown to be protective. This study examined predictors of participating in a telephone-based social connectedness intervention for socially isolated older adults.

**Methods:**

Data was obtained from a social connectedness intervention that paired students with older adults. Eligible participants included Houston area older adults, 65 years or older, enrolled in Medicare Advantage plans. Eligible participants were contacted telephonically and asked to complete the 3-item UCLA Loneliness Scale. Those who screened positive for loneliness were invited to participate in the social connectedness intervention. Logistic regression models, that accounted for sociodemographic, clinical and functional indices, were used to identify predictors of participation.

**Results:**

Females (OR: 1.23, 95% CI: 1.04-1.45), and racial/ethnic minorities (African American OR: 1.39, 95%CI: 1.16-1.68; Hispanic OR: 1.43, 95%CI: 1.04-1.99) were more likely to participate in a telephone-based social connectedness intervention. Older adults with a disability (OR: 2.37, 95%CI: 1.96-2.87), higher CMS Hierarchical Condition Category risk scores (OR: 1.15, 95%CI: 1.06-1.25) and those who reported having 1+ social needs (OR: 1.38, 95%CI: 1.14-1.66) were more likely to participate. Of all Charlson Comorbidity Index flags examined, diabetes was the single strongest predictor of participation (OR=2.49, p=0.02). Older adults who reported functional comorbidities for anxiety and depression were also more likely to participate.

**Conclusion:**

Telephone-based social connectedness interventions can reach vulnerable older adults with clinical and social needs, and can be useful in addressing racial/ethnic health equity gaps in socially isolated older adults.

